# Combined DNA/RNA Amplicon Sequencing and Metatranscriptomics Reveals Microbial‐Driven Nutrient Transformations and Core Taxa in Agriculturally Impacted Sediments

**DOI:** 10.1111/1758-2229.70205

**Published:** 2025-09-27

**Authors:** Nicholas W. Falk, Ian G. Droppo, Ken G. Drouillard, Christopher G. Weisener

**Affiliations:** ^1^ Flinders University Bedford Park Australia; ^2^ Great Lakes Institute for Environmental Research University of Windsor Windsor Canada; ^3^ Canada Centre for Inland Waters, Environment and Climate Change Canada Burlington Canada

**Keywords:** agriculture, DNA vs. RNA, Great Lakes watersheds, metatranscriptomics, nitrogen, phosphorus, sediments

## Abstract

Chronic non‐point nitrogen (N) and phosphorus (P) loads reshape sediment microbial biogeochemical cycling in headwater systems, altering ecosystem function. This study integrates DNA and RNA amplicon sequencing with metatranscriptomics to examine microbial taxonomic and functional responses to nutrient inputs in lower Great Lakes watersheds, focusing on N, P, and sulphur (S) metabolism. RNA‐based taxa showed a stronger correlation with metabolic functions than DNA‐based taxa, highlighting RNA‐based approaches as valuable tools for assessing active microbial responses to nutrients. Site‐specific analyses revealed distinct microbial metabolic profiles linked to watershed fertiliser sources and seasonal variation. Inorganic fertiliser inputs were associated with tightly coupled N reduction and sulphur oxidation, driven by differential expression of dissimilatory nitrate reduction to ammonia (DNRA) and *sox* genes. In contrast, a manure‐amended site exhibited elevated nitrosative stress and sulphur assimilation pathways, consistent with detection of ammonia‐oxidising genera. The low‐impact reference site demonstrated intermediate functional turnover, enhanced nitrogen fixation, and the highest microbial diversity, suggesting greater ecosystem resilience. Seasonally, functional turnover increased in fall, with fewer shared core taxa across sites compared to summer. These findings highlight the impact of chronic nutrient enrichment on site‐specific microbial adaptations and underscore the importance of temporal dynamics in assessing freshwater sediment microbial communities.

## Graphical abstract

Excess loads of nitrogen and phosphorus in agriculturally impacted watersheds alter microbial metabolism in sediments. A multi‐omic investigation found nitrate reduction coupled to sulphur oxidation was prominent where inorganic fertilisers were dominant, whilst a manure‐impacted site revealed biomarkers of nitrosative stress due to incomplete microbial elimination of reactive nitrogen species.

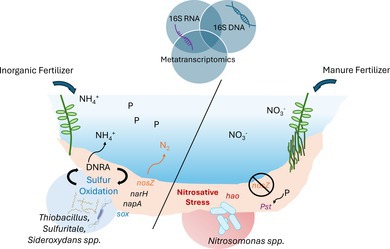

## 1 Introduction

Nutrient runoff from agricultural landscapes is recognised as a leading cause of degraded river water quality worldwide (Newcomer Johnson et al. 2016). Nitrogen (N) and phosphorus (P) sourced from both inorganic (i.e., chemical‐based) and organic (i.e., manure‐based) fertilisers can impair surface waters and sediments and lead to eutrophication, anoxia, and disruption of ecosystem services (Jenny et al. 2020; Le Moal et al. 2018). Watersheds of the lower Great Lakes of North America face these non‐point nutrient threats due to the multiple and combining pressures from intensive row crop agriculture and livestock operations in the region (Singh et al. 2023). Assessment and prediction of N and P transformations in these systems are vital to effectively combat the detrimental effects of nutrients on receiving waterbodies (Kelly et al. 2021; Stutter et al. 2021).

Microbial communities in river sediments are paramount in the biogeochemical cycling of N and P and are vital to ecosystem balance. Denitrifying bacteria remove excess N from aquatic systems through the conversion of nitrate (NO_3_^−^) and nitrite (NO_2_^−^) to N_2_ gas, whilst dissimilatory nitrate reducers and nitrifying bacteria cycle and retain N in the form of ammonia (NH_3_), which can be utilised by other organisms (Huang et al. 2018). In addition, N‐fixation is microbially mediated and necessary for the assimilation and biosynthesis of multiple life‐sustaining compounds in N‐limited systems. P is essential to many microbial processes and is a vital component of nucleic acids, membranes, and ATP. Thus, P is often considered the limiting nutrient for primary production in impacted aquatic systems (Records et al. 2016). Microbes can uptake and store P in large quantities in conditions of limitation (McMahon and Read 2013; Sanz‐Luque et al. 2020), and gene‐level control of these transformations is informative in P cycling and transport modelling. Sulphur (S) is another principal biological macronutrient, with sulphate (SO_4_^2−^) serving as a microbial terminal electron acceptor in oxygen and nitrate‐deficient sediments and is coupled with the bioavailability of P and iron (Fe) (Lamers et al. 2012) and linked to N‐reduction (Li et al. 2022). Characterising the diversity and function of sediment microbial communities is thus central to the understanding of nutrient transformations and fate in agriculturally impacted environments where N and P are in excess of natural background concentrations (Horton et al. 2019).

Culture‐independent genomic techniques that sequence DNA and RNA from sediment samples can inform on the variety of microbes present (through 16S ribosomal amplicon sequencing, for example) and the genes responsible for macronutrient reactions (through shotgun sequencing of total extractable nucleic acids) (Febria et al. 2010; Gibbons et al. 2014; Jewell et al. 2016; Zemskaya et al. 2021). However, a limited number of studies have sought to combine amplicon and shotgun approaches to find consensus in microbial roles in nutrient‐stressed freshwater environments. Metatranscriptomics and amplicon sequencing of microbial communities in marine sediments have identified site‐specific and temporal trends in nitrogen cycling, revealing *Nitrosopumilus* as an active nitrifier where N and organic carbon inputs were high (Marshall et al. 2021, 2023). Elsewhere, taxonomic analysis of ribosomal RNA (rRNA) and functional analysis of messenger RNA (mRNA) were used to examine planktonic microorganisms across European lakes with varying phosphate levels. The findings suggest that whilst taxon diversity differed between lakes, leading to distinct ecological roles, core functions remained consistent (Grossmann et al. 2016). Multiple studies have also highlighted the significance of measuring both DNA‐based and RNA‐based taxonomy to assess the total and active microbial communities present, respectively, citing that RNA‐based methods may better represent the functionally relevant microbial guilds (Broman et al. 2021; Goltsman et al. 2015; Jia et al. 2020; Loeppmann et al. 2018; Meyer et al. 2019). Although metatranscriptomic analysis can incorporate taxonomic identification, the common practise of depleting rRNA from samples (to increase the % yield of mRNA) as well as the lack of specific functional gene‐to‐taxa databases of freshwater‐derived microorganisms results in a low taxonomic resolution from mRNA alone in these study systems. Thus, using and co‐interpreting taxonomic and functional methods enables an improved view of microbial community diversity and function in aquatic systems and can reveal more relevant taxa/genes corresponding to sediment nutrient cycling. Yet there is a lack of such applications to non‐point source nutrient‐affected headwaters.

Building on this established knowledge of microbially driven nutrient cycling and omic analyses, we propose several hypotheses:

1. RNA‐based taxa datasets will correlate more strongly with metatranscriptomic datasets than DNA‐based taxa, as RNA better represents functionally active species.
2. Agriculturally impacted sites will differ from reference sites both spatially and temporally in microbial community structure and function.
3. Agriculturally impacted sites will exhibit lower microbial gene expression related to phosphorus uptake and higher expression of genes for nitrogen removal (e.g., denitrification) driven by fertiliser‐enriched nutrient availability, compared to reference sites.
4. Nitrogen fixation will be more pronounced at low‐impact reference sites, where bioavailable nutrients are not oversupplied by fertiliser runoff.

To test these hypotheses, we co‐extracted DNA and RNA from sediments in three Great Lakes headwaters influenced by distinct non‐point nutrient sources: chemical fertiliser amendment, manure fertiliser amendment, and a low‐impact reference site, sampled across two seasons (summer and fall). 16S amplicon sequencing of both DNA and RNA was conducted to distinguish total versus active microbial taxa, followed by RNA‐seq metatranscriptomics to assess functional genes associated with nitrogen (N), phosphorus (P), and sulphur (S) metabolism. Comparative analyses examined whether DNA‐based or RNA‐based amplicon data correlated more strongly with nutrient‐cycling genes. We then identified the most active and abundant taxa and analysed differentially expressed functional genes across sites to build an understanding of microbial responses to specific nutrient impacts.

## 2 Methods and Materials

### 2.1 Sampling Locations

Three river segments in southern Ontario (Canada) were sampled in the summer and fall of 2020; Big Creek (BC), Nissouri Creek (NC), and the Saugeen River (SR). Watercourses were selected based on the prevailing watershed agricultural practise. In brief, BC and NC are within agricultural catchments (> 70% agricultural landcover), with BC characterised by inorganic fertiliser application and NC characterised by a manure/inorganic fertiliser mix (Table S1), whilst the SR site is in a higher forested catchment (< 35% agricultural landcover) representing a pseudo‐natural reference site to contrast against the other two sites. BC and NC are headwaters of the Thames River, which is a major accumulator and transporter of nutrients to Lake St. Clair and the Detroit River of the Great Lakes Huron–Erie Corridor system (Bocaniov et al. 2019). The SR sampling site is within an upstream region of the river ~140 km from its outlet to Lake Huron and serves as a low nutrient‐impact system.

The region has a humid continental climate with cold, snowy winters, mild autumns, and warm, humid summers. Average daily summer temperatures are 21.9°C, 19.6°C, and 17.8°C at BC, NC, and SR, respectively, whilst fall averages are 11.9°C, 10.1°C, and 9.4°C. Rainfall patterns differ slightly amongst sites: BC receives most precipitation in May–June (mean total 86 mm), whereas NC and SR peak in August–September with 92 mm and 70 mm, respectively. Summer and fall rainfall during the study period was consistent with long‐term averages (Government of Canada 2022).

Sampling dates were chosen to avoid rainfall effects on stream dynamics. Specifically, rainfall in the 2 days prior to sampling remained within one standard deviation of the seasonal mean (summer: June 1–August 30; fall: September 1–November 30). This ensured stream conditions reflected typical seasonal patterns rather than being influenced by runoff or dilution from heavy rainfall events.

### 2.2 Bed Sediment and Surface Water Sampling

Bed sediment was collected in triplicate at each site with a sterile trowel to shovel sediment into sterile, wide mouth high‐density polyethylene (HDPE) containers. Sediment was collected at the sediment–water interface (SWI) to limit exposure to the overlying water column. From each triplicate HDPE container, smaller sediment subsamples of 4–5 g were transferred into cryotubes in the field using sterilised spatulas and immediately flash‐frozen in liquid nitrogen in a 4 L field dewar. Cryotubes were filled to 3/4 to avoid rupturing when flash freezing. Sites were wadable and did not exceed 0.7 m in depth.

Surface water for assessing nutrient levels was collected in triplicate from the midsection of each watercourse segment in 250 mL HDPE bottles at a depth of 20 cm below the surface. Water was filtered in the field through 0.22 μm cellulose‐acetate filters for dissolved analytes. Water samples were kept on ice until storage at −4°C until analysis (up to 1 week). General water quality parameters (temperature, dissolved oxygen, and pH) were recorded via a YSI EXO2 Multiparameter Water Quality Sonde. For nutrient analytes, N chemistry (NO_3_ (nitrate‐nitrogen), NO_2_ (nitrite‐nitrogen), NH_4_, Total Organic Nitrogen (TON)), P chemistry (soluble reactive P (SRP), Total P (TP)), and SO_4_ were measured on a Thermo Gallery Plus Beermaster Autoanalyser, with Total N (TN) measured on a Shimadzu TOC‐L/TNM‐L CPH Model Analyser according to EPA standards. Analyses were performed at the University of Alberta's Natural Resources Analytical Laboratory (NRAL).

### 2.3 DNA and RNA Amplicon Sequencing for Microbial Taxonomic Analysis

#### 2.3.1 Extraction and Sequencing

Sediment samples were co‐extracted for total RNA and DNA using the RNeasy PowerSoil Total RNA (Qiagen, Hilden, Germany) and RNeasy PowerSoil DNA Elution Kits (Qiagen, Hilden, Germany). Bed sediments were processed per the manufacturer's instructions using 2 g of thawed sediment per extraction. An aliquot of the total RNA fraction was DNA‐depleted using the RapidOut DNA Removal Kit (Thermo Scientific) and converted to copy DNA (cDNA) using the High‐Capacity RNA‐to‐cDNA kit (Applied Biosystems, Waltham, Massachusetts, United States). All DNA and cDNA samples were subjected to Polymerase Chain Reaction (PCR) to amplify an approx. 300 base pair (bp) segment of the hypervariable 16S prokaryote rRNA gene. Forward primer V5F (5′‐ATTAGATACCCNGGTAG‐3′) and reverse primer V6R (5′‐CGACAGAGCCATGCANCACCT‐3′) were used in the PCR reaction with 1 μL of DNA or cDNA, with additional reagents and reaction parameters specified previously (Falk et al. 2022). Resulting amplicons were visualised on 1% agarose gels to confirm sequence length, followed by a modified bead cleaning based on the Agencourt AMPure XP PCR purification protocol to remove fragments < 50 base pairs. A second PCR was performed to attach unique barcode and adapter nucleotides to each sample for sequencing in multiplex. Samples were then combined and excised through a final 2% agarose gel extraction (QI‐Aquick Gel Extraction KitTM, Qiagen, Hilden, Germany), normalised for concentration on an Agilent 2100 Bioanalyzer, and sequenced on the University of Windsor's Ion GeneStudio S5 Sequencer (ThermoFisher, Waltham, Massachusetts, United States) with output in single‐end FASTQ file format. A total of 48 sediment samples were sequenced, with demultiplexed raw data submitted to the Sequence Read Archive under accession number PRJNA927737 (Table S2).

#### 2.3.2 16S Microbial Community Profiling

DNA‐ and RNA‐based sample FASTQ files were processed through the Quantitative Insights into Microbial Ecology (QIIME) pipeline, QIIME2 ver. 2021.4. After filtering (Callahan et al. 2016) and alignment (Katoh et al. 2002), samples were rarefied to 10,000 sequences for beta diversity pairwise comparisons using the q2‐phylogeny plugin (Faith 1991; Lozupone et al. 2007; Lozupone and Knight 2005). Microbial taxonomy was assigned to amplicon sequence variants (ASVs) using the SILVA ribosomal RNA database (Silva release 132_99_16S) (Quast et al. 2013) with a Naive Bayes classifier (Bokulich et al. 2018) trained on sequences extracted using the primers specified. The resulting hierarchical taxonomy‐by‐sample table was used for microbial community analysis and visualisation, with analysis done at the genus level.

### 2.4 mRNA Sequencing and Metatranscriptomics

Bed sediment samples were analysed for total prokaryotic gene function via mRNA metatranscriptomics. After extraction, as described in Section 2.3.1, aliquots of RNA were checked for purity and quantity on an Agilent 2100 Bioanalyzer and sent to the Centre d'expertise et de services Génome Québec (https://cesgq.com/home) for bacterial rRNA depletion (Ribo‐Zero rRNA removal kit), reverse transcription to cDNA, library preparation, and shotgun sequencing on an Illumina NovaSeq 6000 System (Montréal (Québec), Canada). Sequence reads were processed through the MG‐RAST (Metagenomics Rapid Annotations using Subsystems Technology) pipeline (Meyer et al. 2008). Details of preprocessing, dereplication, and gene calling can be found in Keegan et al. (2016). Briefly, clustering of proteins was performed at 90% similarity using uclust (Edgar 2010), with protein identification and annotation mapping done via sBLAT and using the M5NR database. Annotations were selected using the ‘Representative Hit’, classification, with functional profiles of feature hits across samples produced using the hierarchical SEED Subsystems classification (Overbeek et al. 2014). Subsystems uses a four‐level hierarchy organisation, with functional prediction increasing in specificity as levels increase. The highest level (L1) represents a broad functional category containing many features surrounding a central cellular process, whilst the lowest level (L4, or ‘function‐level’) produces a functional assignment (often a protein‐coding gene) specific to a single reaction. Detailed site and seasonal analysis were restricted to functions within the L1 categories ‘Nitrogen Metabolism’, ‘Phosphorus Metabolism’, and ‘Sulphur Metabolism’. A total of 16 sediment samples were produced, with metatranscriptomic libraries available through MG‐RAST under the IDs outlined in Table S3.

### 2.5 Microbial Community and Functional Analysis

#### 2.5.1 Mantel Tests of Microbial Taxonomy (DNA and RNA) vs. Function

**FIGURE 1:**
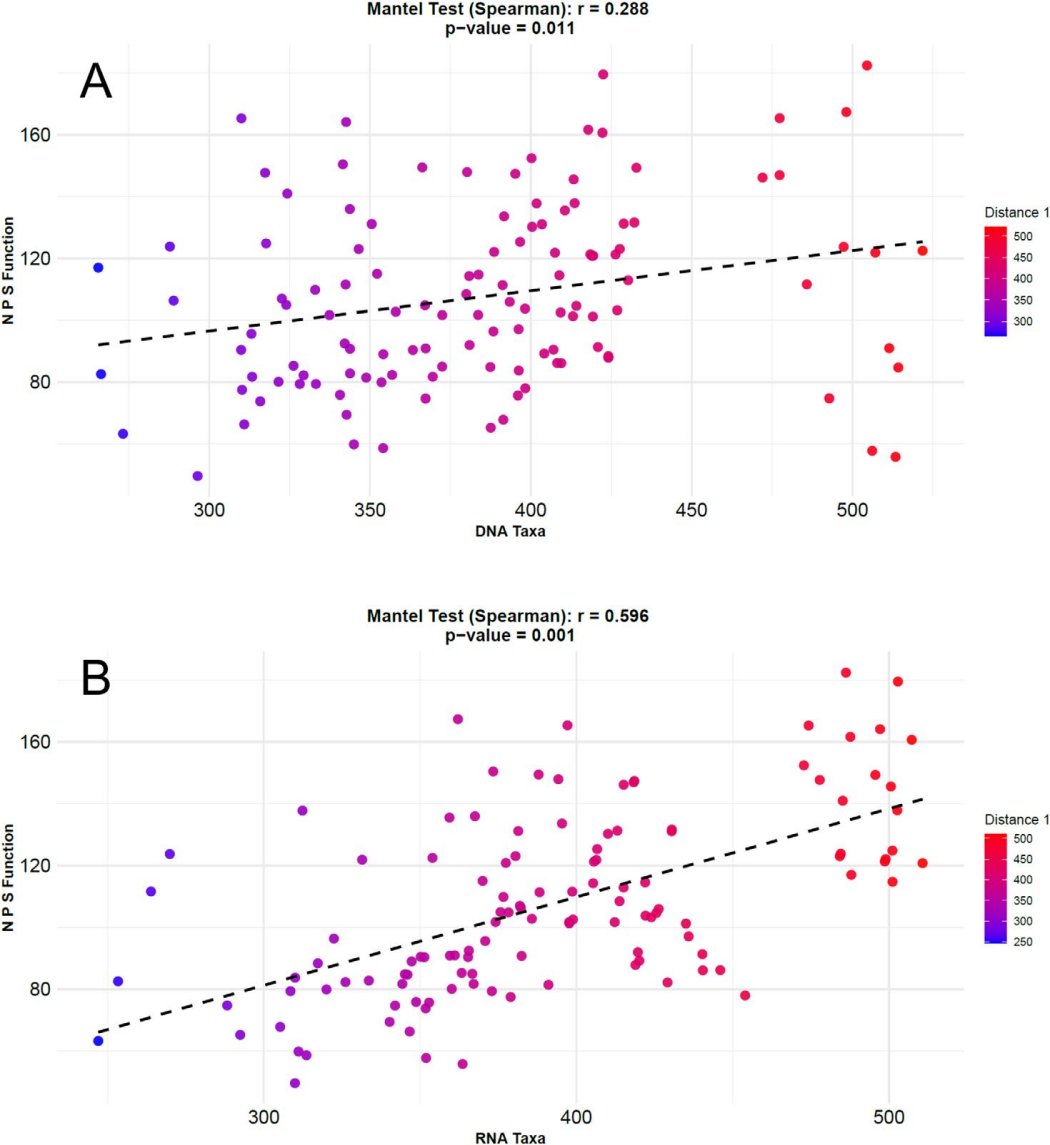
Mantel test results comparing sample distances between16S amplicon taxonomy and Nitrogen (N), Phosphorus (P), and Sulphur (S) metabolism functions. (A) DNA‐based 16S amplicon data vs. functions, (B) RNA‐based 16S amplicon data vs. functions. Points represent the comparison of Euclidean distances between paired amplicon samples (*x*‐axis values) and paired metatranscriptomic samples (*y*‐axis values).

DNA‐ and RNA‐based taxa‐by‐sample count tables at the genus level were compared to the N, P, and S metatranscriptomic L4 function‐by‐sample count table via mantel tests to assess the degree of correlation of the total (DNA) and active (RNA) microbial community composition to microbial community nutrient cycling functions. As the mantel test required matching sample numbers between matrices, the amplicon dataset (*n* = 48 samples) was first reduced by random exclusion of samples to match the sample numbers in the metatranscriptomics dataset (*n* = 16). The count tables were then normalised by cumulative sum scaling (CSS) (Paulson and Bravo 2013), converted to distance matrices using Euclidean distance, and subjected to a mantel test (R vegan package) using the Spearman correlation method with *r* ^2^ and *p*‐values calculated. Mantel test results indicated a stronger correlation between RNA‐based taxa and N, P, and S functions (*r* ^2^ = 0.596, *p* = 0.001) than DNA‐based taxa to N, P, and S functions (*r* ^2^ = 0.288, *p* = 0.011) (Figure 1); thus, RNA‐based taxa were weighted more strongly in additional analysis of core active taxa (Section 2.5.2).

#### 2.5.2 Methodology for Identification of Core–Active Taxa

The core‐active taxa for each sampling time and site were identified by leveraging presence/absence, significance in RNA‐amplicon data via Linear discriminant analysis Effect Size Estimation (LEfSe) (Segata et al. 2011), and overall relative abundance. In brief, genus count tables were first filtered for taxa detected in both DNA and RNA (i.e., a count of at least 1 in each dataset) as a conservative measure of presence in samples, and then LEfSe was used to sort the taxa by their significance in RNA over DNA (*p* < 0.05) as RNA‐based taxa showed a stronger correlation to nutrient cycling functions (Section 2.5.1). These significant taxa were then ranked, highest to lowest, by their average abundance in RNA and DNA. These were rendered as the core‐active taxa, as they were detected in both amplicon datasets, but with greater detection from RNA and were highly abundant.

#### 2.5.3 Site and Season Differential Microbial Function Within Nitrogen, Phosphorus, and Sulphur Metabolism

The functions of nitrogen (N), phosphorus (P), and sulphur (S) subsystems were pairwise compared across season (within site) and pairwise by location (between site by season) by differential expression analysis using DESeq2 (Love et al. 2014). The identified differential functions were then tallied into their L3 subsystem categories for visualisation and organisation. For three‐way site comparisons of functions in summer and fall, the differentially expressed functions as identified by DESeq2 were further analysed by LEfSe and presented within their detailed pathways of N, P, and S metabolism. This additional analysis presents the most contrasting functions between sites, as normalised across the three groups. DESeq2 and LEfSe results are summarised in Tables S3–S7.

### 2.6 Scanning Electron Microscopy

Scanning electron microscopy (SEM) was performed on selected sediment samples. Collected sediment was kept at 4°C after collection and visualised the following day. Sediment was adhered to microscope stubs with carbon tape and allowed to air for 1 h and then examined using a FEI Quanta 200F ESEM under low vacuum at ranges of 5–17 kV.

## 3 Results

### 3.1 Nutrient Loads Across Sites

Water quality parameters across sites for summer and fall 2020 are displayed in Table 1. Total Nitrogen (TN) was higher at agricultural sites and greater in the summer than the fall, with Nissouri Creek (NC) defined by higher NO_3_ concentrations and Big Creek (BC) defined by higher NH_4_. Total phosphorus (TP) and soluble reactive phosphorus (SRP) concentrations were also higher at the agricultural sites, with BC exhibiting greater levels in the summer (0.16 and 0.05 mg L^−1^ for TP and SRP, respectively) and NC higher levels in the fall compared to summer (0.46 and 0.17 mg L^−1^). SO_4_ was higher in surface waters at the agriculturally impacted sites, and higher in summer than in fall.

**TABLE 1:** Seasonal water quality parameters for Big Creek, Nissouri Creek, and the Saugeen River.

Parameter	Season	Big creek (BC)	Nissouri creek (NC)	Saugeen river (SR)
Temp (°C)	Summer	26 (0.1)	20.4 (0.2)	24.0 (0)
	Fall	13.8 (0)	10.1 (0)	9.7 (0)
pH	Summer	8.1 (0.1)	8.6 (0.1)	8.5 (0)
	Fall	8.3 (0.01)	8.8 (0.03)	8.8 (0.03)
DO (mg L^−1^)	Summer	9.01 (0.03)	10.46 (0.08)	10.89 (0.05)
	Fall	10.55 (0.02)	12.34 (0.03)	11.39 (0.01)
NO_3_^−^ (mg L^−1^)	Summer	1.8 (0.14)	4.65 (0.07)	0.2 (0)
	Fall	0.35 (0.005)	1.01 (0.004)	0.23 (0.003)
NO_2_^−^ (μg L^−1^)	Summer	bdl	bdl	bdl
	Fall	3.1 (0.19)	1.5 (0.3)	0.9 (0.05)
NH_4_^+^ (μg L^−1^)	Summer	130 (0)	30 (0)	20 (0)
	Fall	36.3 (14)	16.4 (3)	24.5 (3)
TON (mg L^−1^)	Summer	0.95 (0.07)	0.3 (0)	0.4 (0.14)
	Fall	0.35 (0.005)	1.02 (0.004)	0.23 (0.003)
TN (mg L^−1^)	Summer	2.75 (0.21)	4.95 (0.07)	0.6 (0.14)
	Fall	0.94 (0.02)	1.21 (0.02)	0.62 (0.01)
SRP (mg L^−1^)	Summer	0.05 (0.004)	0.02 (0.004)	0.007 (0.005)
	Fall	0.08 (0.0006)	0.17 (0.004)	0.02 (0.004)
TP (mg L^−1^)	Summer	0.16 (0.01)	0.06 (0)	0.005 (0)
	Fall	0.19	0.46	0.05
SO_4_^2−^ (mg L^−1^)	Summer	32 (0)	30 (0)	5 (0)
	Fall	19.4 (0.2)	8.4 (0)	1.7 (0)

*Note:* Brackets represent standard deviations.

Abbreviations: bdl, below detection limit; DO, dissolved oxygen; NH_4_^+^, ammonium‐nitrogen; NO_2_^−^, nitrite‐nitrogen; NO_3_^−^, nitrate‐nitrogen; SRP, soluble reactive phosphorus; TN, total nitrogen; TON, total organic nitrogen; TP, total phosphorus.

### 3.2 Taxa Correlations and Core‐Active Taxa

Mantel test results showed a stronger correlation of RNA‐based taxa to N, P, and S metabolism functions (Methods Section 2.5.1, Figure 1). Thus, RNA‐based taxa were weighted more strongly in additional analysis of core‐active taxa (Methods Section 2.5.2).

**FIGURE 2:**
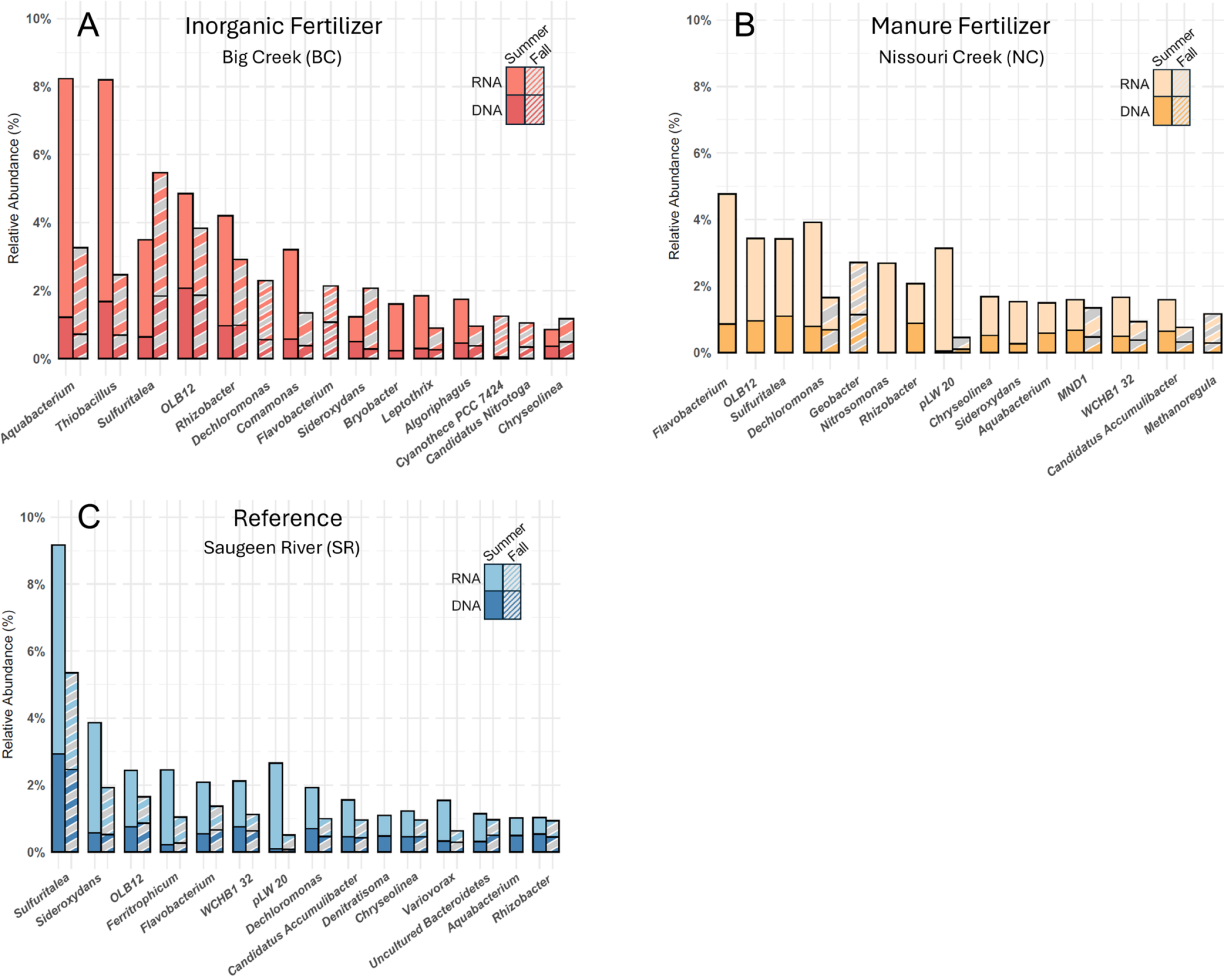
The top 15 core‐active genera by relative abundance (%) across sites identified by leveraging DNA‐based and RNA‐based amplicon sequencing data.

For core‐active taxa, 34 genera were identified at BC (15 in Summer, 19 in Fall), 55 at NC (20 in Summer, 35 in Fall), and 68 at SR (41 in Summer, 27 in Fall). The top 15 most abundant core‐active taxa for each site are displayed in Figure 2 by season and by detected abundance in both DNA and RNA. BC was most abundant in *Aquabacterium*, *Thiobacillus*, *Sulfuritalea*, *OLB12*, and *Rhizobacter*; NC in *Flavobacterium*, *OLB12*, *Sulfuritalea*, *Dechloromonas*, and *Geobacter*; and SR in *Sulfuritalea*, *Sideroxydans*, *OLB12*, *Ferritrophicum*, and *Flavobacterium*. Across the three sites, the shared core‐active taxa in Summer were *Aquabacterium*, *OLB12*, *Rhizobacter*, *Sulfuritalea, Sideroxydans*, and *Chryseolinea*, and in Fall were *Dechloromonas*, *Leptothrix*, and *Candidatus Accumulibacter*.

### 3.3 Metatranscriptomic Overview and Spatial and Temporal Expression in N, P, and S Metabolism

**FIGURE 3:**
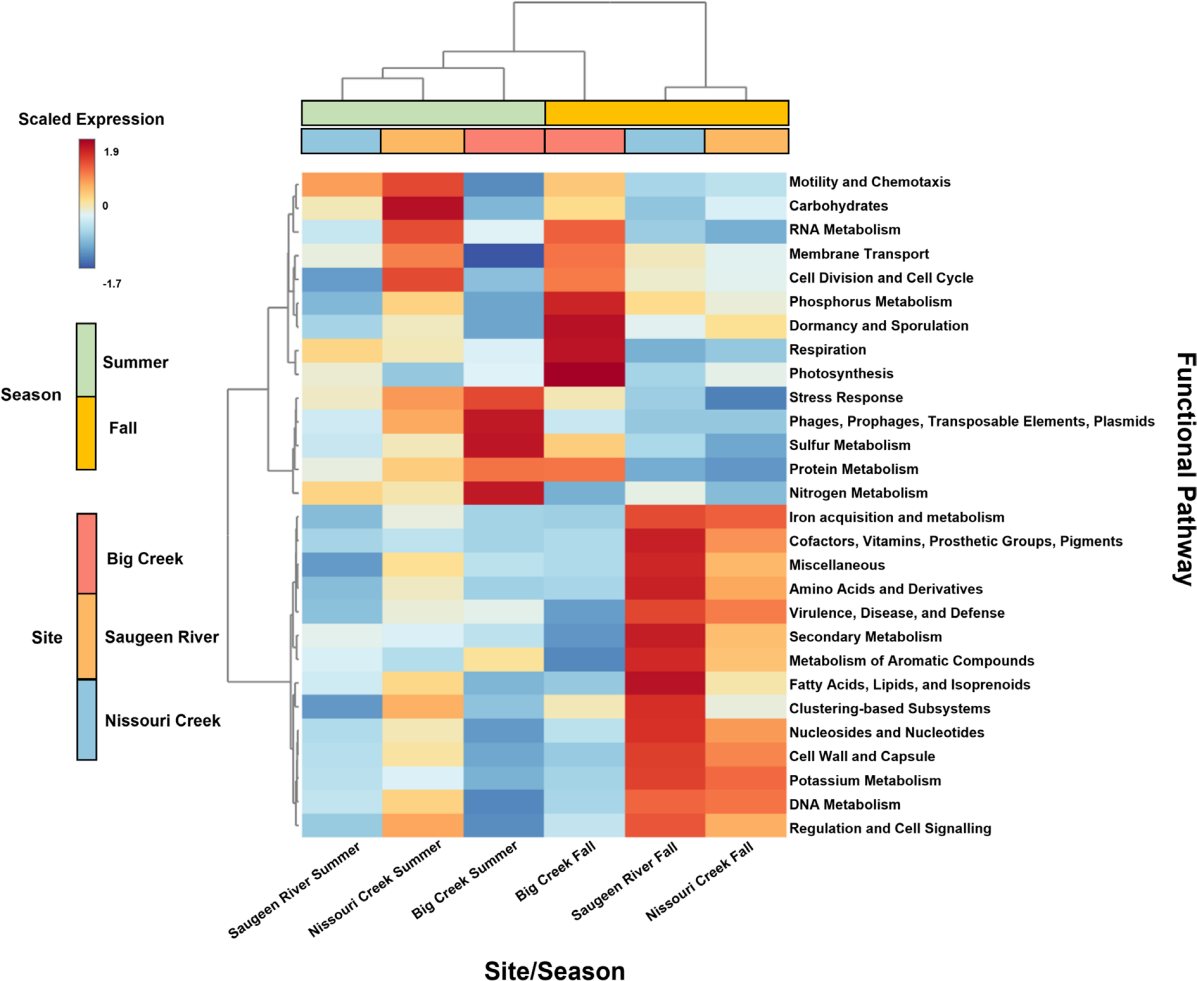
Heatmap of level 1 subsystem pathway expression across site and season.

Figure 3 shows a heatmap of Level 1 functional categories across sample groups. Samples clustered primarily by season, with *Nitrogen Metabolism* and *Sulphur Metabolism* subsystems clustered closely together, and *Phosphorus Metabolism* more separated.

**FIGURE 4:**
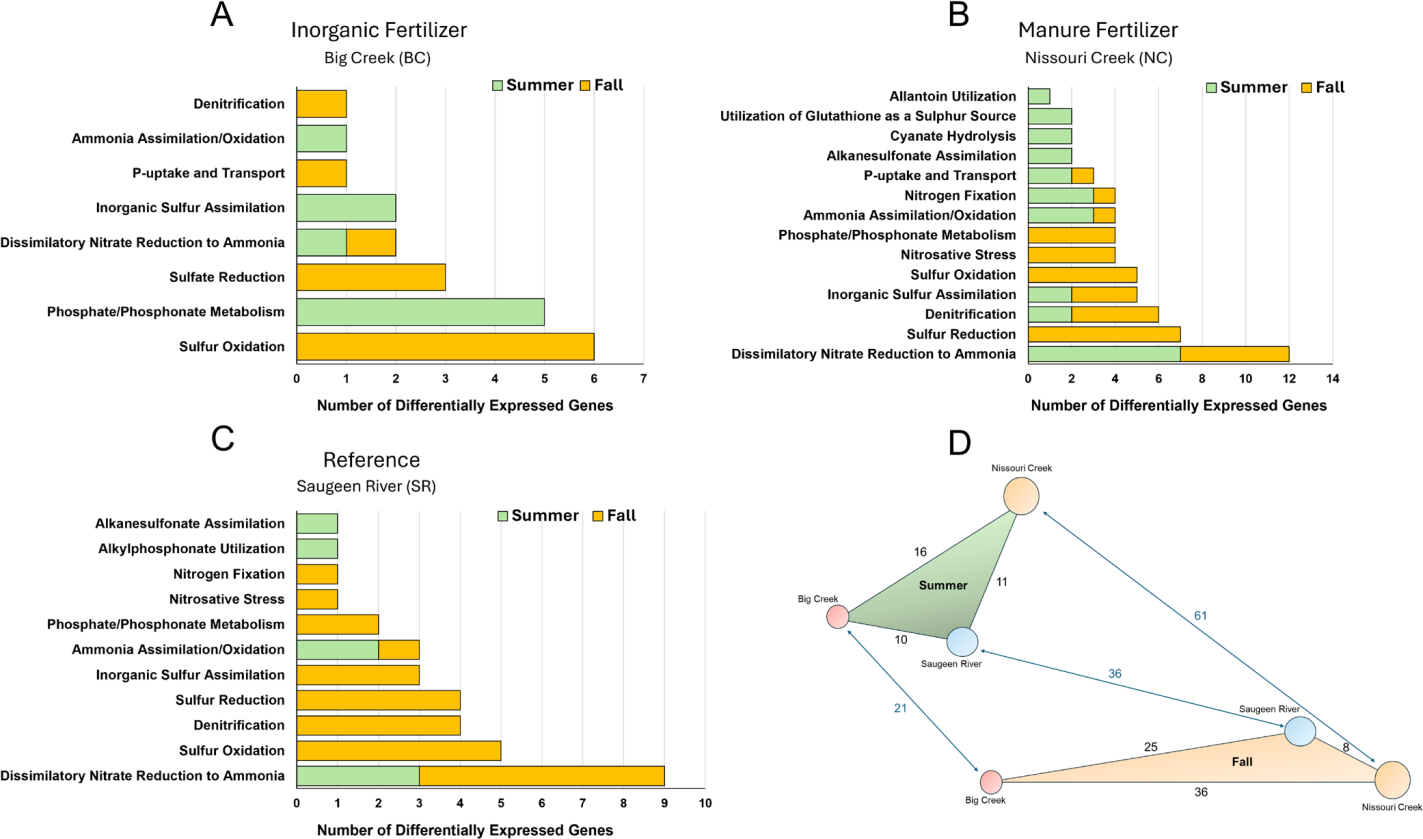
Summary of differences in N, P, and S pathways for summer and fall within sites. (A) Inorganic fertiliser‐impacted site (Big Creek), (B) manure fertiliser‐impacted site (Nissouri Creek), (C) low impact reference site (Saugeen River). (D) conceptual diagram representing the functional differences between sites and seasons. Values represent the number of differentially expressed functions between each group, reflected in the distance between groups.

Seasonal differences in N, P, and S functions within sites are summarised in Figure 4 and Table S6. NC exhibited the greatest number and variety of differentially expressed functions between summer and fall (*n* = 61), with the majority of turnover within *Dissimilatory Nitrate Reduction to Ammonia* (*DNRA*), *Sulphur Reduction*, and *Denitrification*. SR differentially expressed functions were the next greatest (*n* = 34), whilst BC exhibited fewer seasonal differences (*n* = 21), with the most functional turnover within *Sulphur Oxidation*.

**FIGURE 5:**
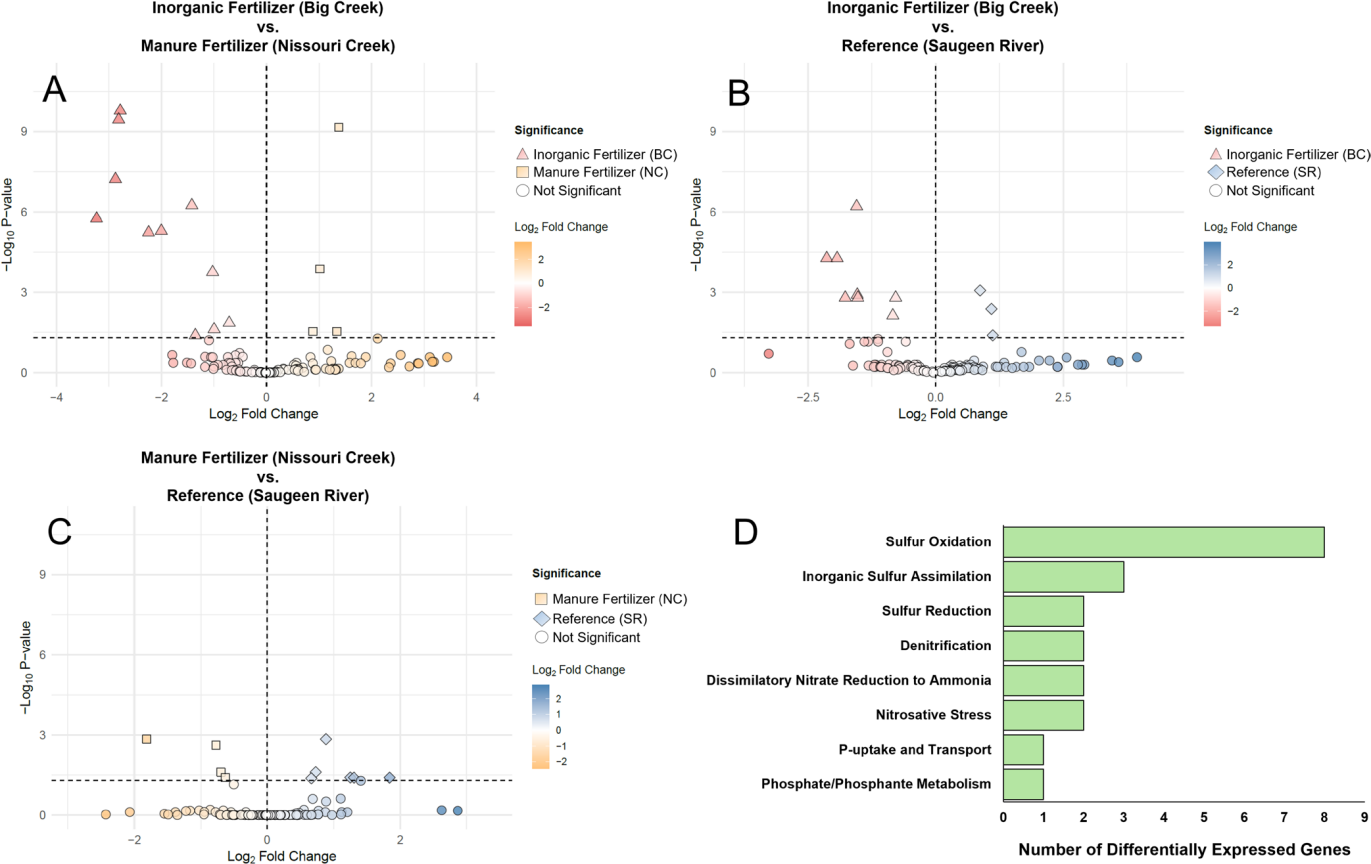
Summer pairwise differential expression of Nitrogen (N), Phosphorus (P), and Sulphur functions between sites. (A) Inorganic vs. manure, (B) inorganic vs. reference, (C) manure vs. reference. (D) Tally of differentially expressed functions by pathway between all sites in summer.

**FIGURE 6:**
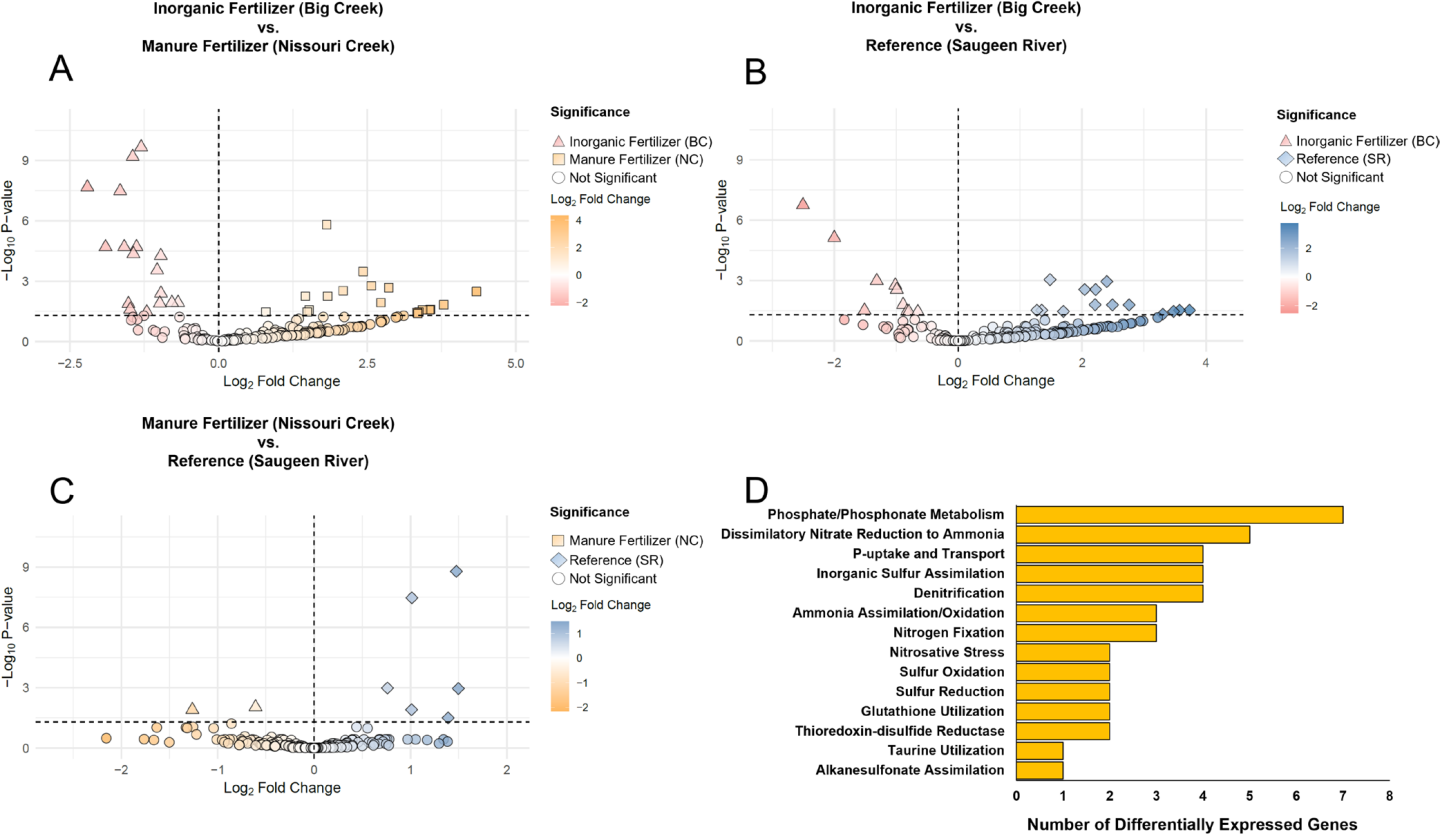
Fall pairwise differential expression of nitrogen (N), phosphorus (P), and sulphur functions between sites. (A) Inorganic vs. manure, (B) inorganic vs. reference, (C) manure vs. reference. (D) Tally of differentially expressed functions by pathway between all sites in fall.

Pairwise site differences in N, P, and S functions are summarised for summer and fall in Figures 5 and 6, respectively (complete data in Tables S4 and S5). For summer, BC vs. NC showed the greatest number of differentially expressed functions (*n* = 16) compared to BC vs. SR (*n* = 11) and NC vs. SR (*n* = 10). Genes within *Sulphur Oxidation* accounted for the greatest number of differentially expressed functions (*n* = 8) between sites, followed by *Inorganic Sulphur Assimilation* and *Sulphur Reduction*. For fall, there were more differentially expressed functions between sites, led again by BC vs. NC (*n* = 36), then BC vs. SR (*n* = 25), and NC vs. SR (*n* = 8). There was also a greater variety of genes differentially expressed in the fall, led by functions within *Phosphate/Phosphonate Metabolism* and *DNRA*.

**FIGURE 7:**
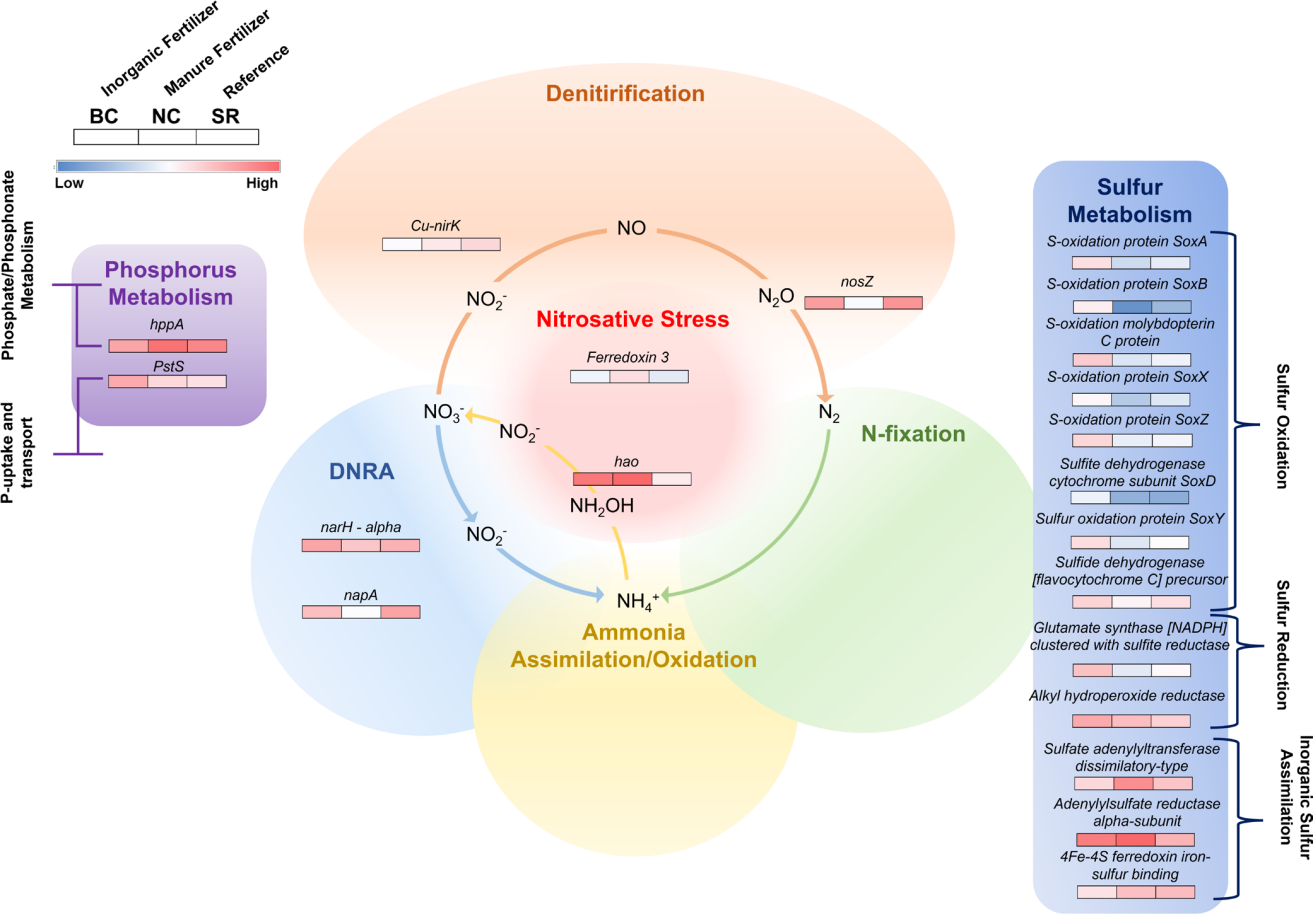
Summer differentially expressed genes within phosphorus, nitrogen, and sulphur metabolism across Big Creek (BC) Nissouri Creek (NC) and Saugeen River (SR) bed sediments. Level 3 subsystem pathway categories are denoted by brackets for phosphorus and sulphur genes. Nitrogen level 3 pathways of denitrification, dissimilatory nitrate reduction to ammonia (DNRA), nitrosative stress, N‐fixation, and Ammonia Assimilation/Oxidation are denoted in the N‐cycling pathway diagram. The scale bar represents normalised expression calculated from LEfSe analysis.

**FIGURE 8:**
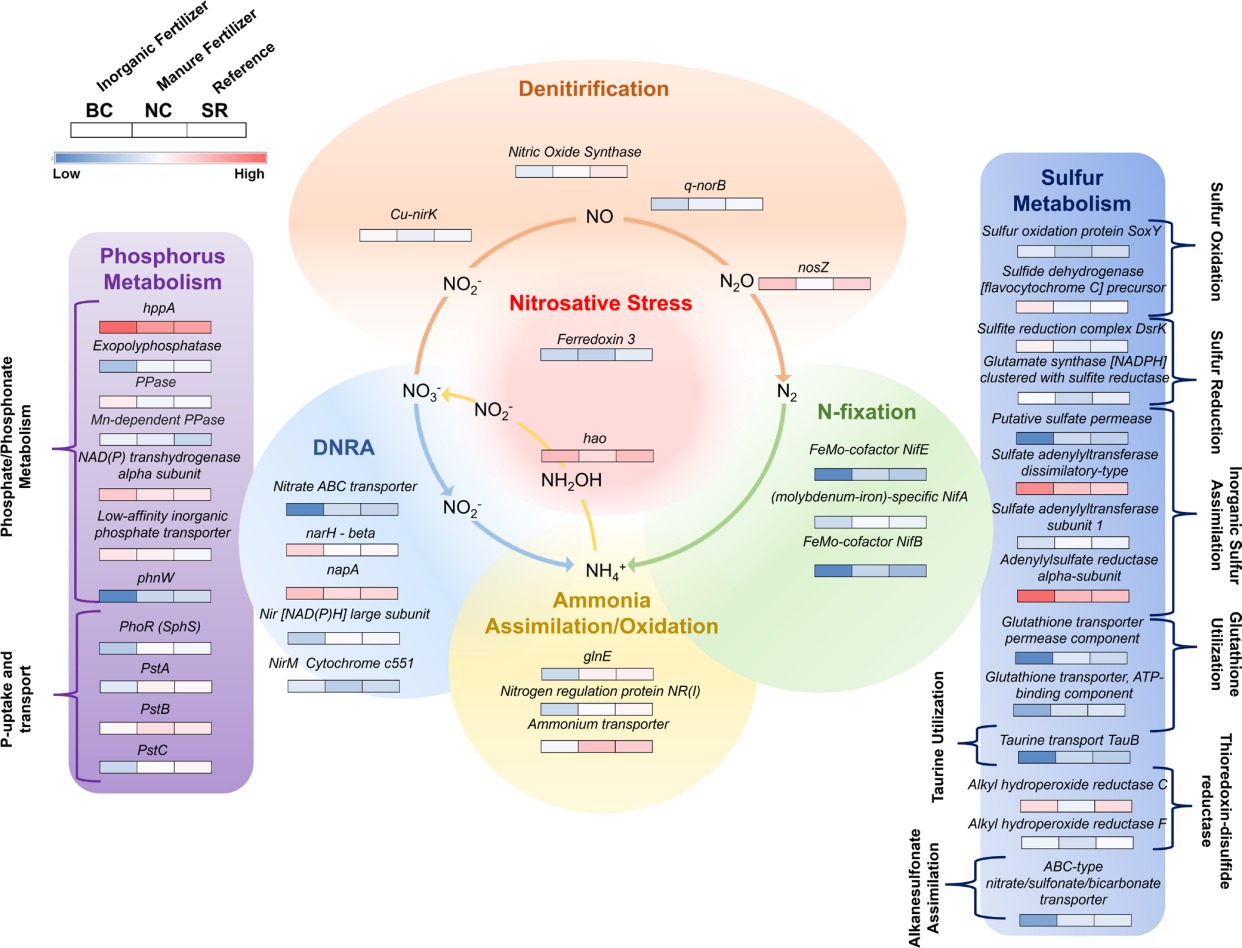
Fall differentially expressed genes within Phosphorus, Nitrogen, and Sulphur metabolism across Big Creek (BC), Nissouri Creek (NC), and Saugeen River (SR) bed sediments. Level 3 subsystem pathway categories are denoted by brackets for phosphorus and sulphur genes. Nitrogen level 3 pathways of denitrification, dissimilatory nitrate reduction to ammonia (DNRA), nitrosative stress, N‐fixation, and ammonia assimilation/oxidation are denoted in the N‐cycling pathway diagram. The scale bar represents normalised expression calculated from LEfSe analysis.

Site‐specific functional gene differences for N, P, and S metabolism are explored further by their higher subsystem pathways in Figures 7 and 8 for summer and fall, respectively, and detailed below (complete data in Tables S7 and S8).

#### 3.3.1 Summer Functional Trends

Within *Nitrogen Metabolism*, BC and SR exhibited significantly greater expression of a membrane‐bound nitrate reductase (*narH*) alpha chain gene involved in the microbial conversion of nitrate to nitrite, as well as greater expression of a periplasmic nitrate reductase (*napA*), both operating within *DNRA*. A Ferredoxin 3 gene and hydroxylamine reductase (*hao*) classified under *Nitrosative Stress* were significantly more abundant at NC, with *hao* also expressed more at BC than the SR. Nitrous‐oxide reductase (*nosZ*) involved in the final step of denitrification showed greater abundance at BC and SR over NC, and a Cu‐containing nitrite reductase (*nirK*) had greater expression at SR. For P‐metabolism, a pyrophosphate‐energised proton pump gene (*hppA*) was highly expressed across sites and seasons, but significantly more abundant at NC, and a phosphate ABC transporter periplasmic phosphate‐binding protein (*PstS*) showed higher abundance at BC. For S‐metabolism genes, a set of sulphur‐oxidation and reduction genes showed greater abundance at BC than both NC and SR (*SoxA*, *SoxB*, *molybdopterin C*, *SoxX*, *SoxZ*, *SoxD*, *SoxY*, glutamate synthase (with sulphite), and alkyl hydroperoxide reductase). Sulphate adenyltransferase (dissimilatory‐type) and adenylsulfate reductase (alpha‐subunit) were highly expressed across all 3 sites, but greatest at NC.

#### 3.3.2 Fall Functional Trends

Fall sediments showed more inter‐site functional differences than summer. *DNRA* was the main pathway showing differences within Nitrogen Metabolism functions, with *narH* (beta chain) and *napA* exhibiting greater expression at BC, and a nitrate ABC transporter and nitrite reductase *nir* (large subunit, *nir*B) being expressed higher at NC and SR. Under *Nitrosative Stress*, *hao*, which is also involved in ammonia transformations, was highly expressed across all sediments, but lowest at Nissouri Creek. Ferredoxin 3 was lower expressed at NC than BC and SR. The *Denitrification* pathway showed a higher abundance of *nosZ* at BC and SR and also shifts in *nirK* and a nitric‐oxide reductase (*norB*, quinol‐dependent), which were most expressed at BC and SR, respectively. Differentially expressed genes involved in *Ammonia Assimilation/Oxidation* included a glutamate‐ammonia‐ligase adenylyltransferase (*glnE*), nitrogen regulation protein NR(l), and an ammonium transporter, all of which showed greater abundance at NC and SR than BC. The *N‐fixation* pathway showed a similar trend to *Ammonia Assimilation/Oxidation*: *nifE*, *nifA*, and *nifB* all had higher expression at NC and SR and less expression at BC.

For *Phosphorus Metabolism*, two primary pathways comprised differences between sites: *Phosphate/Phosphonate Metabolism* and *P‐uptake and Transport*. Within *Phosphate/Phosphonate Metabolism*, *hppa* was again highly expressed across sites, but most abundant at BC, with a similar trend for an inorganic pyrophosphatase (*PPase*) and an NAD(P) transhydrogenase alpha subunit. A Mn‐dependent *PPase* and a low‐affinity inorganic phosphate transporter were higher expressed at BC and NC. An exopolyphosphatase and 2‐aminoethylphosphonate:pyruvate aminotransferase (*phnW*) were more expressed at NC and SR, and less expressed at BC. Within *P‐uptake and Transport*, all significant genes were in higher abundance at NC and the SR, with lower expression at BC. This included *Pst group genes* involved in P‐uptake and a phosphate regulon sensor (*SphS*) and phosphate transport permeases (*PstA*, *PstC*) involved in the high affinity phosphate (PHO) transporter regulon.

For *Sulphur Metabolism*, fewer genes involving sulphur‐oxidation/reduction were differentially expressed across sediments than in the summer. *SoxY*, a sulphide dehydrogenase, *DsrK*, and a glutamate synthase [NADPH] were all more expressed at BC than NC, with intermediate expression at SR. Within *Inorganic Sulphur Assimilation*, sulphate adenyltransferase (dissimilatory‐type) and adenylsulfate reductase (alpha‐subunit) were again highly expressed across all sites, but greatest abundance was measured at BC opposed to NC as seen in the summer. However, a putative sulphate permease showed higher abundance at NC and SR. The remaining S function genes were spread across ‘*Glutathione Utilisation*’, ‘*Taurine Utilisation*’, ‘*Thioredoxin‐disulphide Reductase*’, and ‘*Alkanesulfonate Assimilation*’, and were mostly higher expressed at NC and the SR and in low abundance at BC. The exception was alkyl hydroperoxide reductase F and alkyl hydroperoxide reductase C, which were higher expressed at BC and the SR.

## 4 Discussion

Headwater sediments impacted by chronic non‐point nutrient pollution from agriculture may have altered microbial biogeochemical cycling compared to non‐impacted reference sites. To contrast these, DNA‐ and RNA‐based approaches were used to leverage the whole and active microbial consortium, respectively, and were layered with metatranscriptomic analysis with a focus on N, P, and S metabolism functions.

### 4.1 RNA‐Based Taxa Align With Nutrient Cycling Functions

From Mantel tests, RNA‐derived taxa showed a stronger correlation to N, P, and S functions than DNA‐derived taxa, supporting our hypothesis. In other words, paired RNA amplicon samples that exhibited dissimilarity or similarity in community composition also showed dissimilarity/similarity in the functional metatranscriptomic dataset. Alternatively, DNA‐based amplicon sequencing showed a weaker connection, where paired samples could be similar in their DNA‐based taxa composition but dissimilar in function, and vice versa. Previous studies have reported differences in DNA‐based and RNA‐based amplicon sequenced microbial communities (De Vrieze et al. 2018; Pochon et al. 2017), but this is the first study to our knowledge to quantify the community similarity to a subset of paired functional data via metatranscriptomics. This suggests that the utilisation of RNA for amplicon sequencing can be more adequate in assessing microbial taxonomic responses to nutrient loads, whereas DNA approaches may be more generic and thus less sensitive. This may partly explain why previous models have found weak linkages between denitrification rates and DNA‐based functional gene characteristics, with environmental characteristics being a better prediction of N‐cycling patterns (Attard et al. 2011; Graham et al. 2014). However, DNA‐based analyses are valuable in assessing holistic microbial functional potential and fundamental niches and are well suited for a variety of hypothesis testing, such as assessing the resilience of microbiomes over longer timeframes (Barnett and Shade 2024). The approach of weighing RNA more heavily in the identification of core‐active taxa across sites here, however, is supported by mantel test results and observations of how taxa aligned with site‐specific functions are expanded on below.

### 4.2 Seasonal and Site Divergence in Microbial Function by Agricultural Nutrient Inputs

Site water chemistry showed that the river segments within the agriculturally intensive watersheds, Big Creek (BC) and Nissouri Creek (NC), had higher nutrient concentrations than the less‐impacted control site, Saugeen River (SR). In addition, previous research showed total and bioavailable sediment P was significantly higher at BC and NC than SR (Falk et al. 2021), evidence that agricultural activity is associated with higher nutrient loads. Microbial taxonomy and function also showed differences between the agricultural sites and the reference site; however, contrary to our hypothesis, more genes were differentially expressed between the two agricultural sites than either site compared to the reference. For example, when compared between sites, BC and NC had 16 and 36 pairwise differentially expressed functions in the summer and fall, respectively, whilst the reference SR site had at most 11 and 25 differentially expressed functions when compared to the other sites. SR was also intermediate between BC and NC in terms of within‐site seasonal differentially expressed functions. Thus, sites in the agriculturally impacted watersheds appeared more distinct from each other and exhibited greater seasonal differences in sediment microbial nutrient metabolisms compared to the more natural reference site. This has been observed in past research, where impacted environments diverge in microbial function, but in manners that reflect the site‐specific anthropogenic stressors (Cagnon et al. 2017; Falk et al. 2019; Van Gray et al. 2020). Here, the form of fertiliser amendment (either inorganic or a manure/inorganic mix) that characterised the watershed was associated with a divergence of microbial function away from the reference state, but in different manners, which is expanded on in Section 4.4.

This divergence in function was also greatest in the fall, where there were 42 differentially expressed functions between sites compared to 21 in the summer. This was associated with a decrease in the number of shared core‐active taxa across sites, where summer had 6 genera shared, whilst fall had 3. Thus, as sediments became more distinct in function, they shared fewer of the same active taxa, suggesting a connection between functional and taxonomic turnover. Temperature variations and increased precipitation in the fall can shift the headwater environment, and this may explain the greater microbial differences between the sites compared to summer, where conditions are more uniform. For example, increased precipitation in the fall can lead to greater loading of microbial cells into waterways, which increases phylogenetic diversity, whereas stagnant summer conditions homogenise communities and increase microbial equality (i.e., Shannon diversity) (Liu et al. 2023; Zhang et al. 2019), which explains the enhanced functional differences seen in fall in this study. Future studies should consider seasonal effects, which may skew the interpretation of differential function if only a single season is monitored, as has been advised for biotic health indices using benthic invertebrates (Callanan et al. 2008).

### 4.3 Site‐Specific Trends in Microbial Metatranscriptomic Profiles

#### 4.3.1 Big Creek—Microbial Functional Relationships to Inorganic Fertiliser Loads

Nitrate reduction and sulphur oxidation were prevalent metabolic pathways in BC sediments. Both denitrification and DNRA co‐occur to reduce nitrate; however, DNRA appeared more prevalent from the high expression of *narH* and *napA* transcripts. Lower sediment redox increases DNRA over denitrification, as identified previously in sediments (Biessy et al. 2022). DNRA could be contributing to the high concentrations of ammonium detected in surface waters, in addition to ammonium nitrate, urea, or ammonium sulphate sourced from inorganic fertiliser use that characterises the BC watershed. DNRA can be energetically coupled to sulphur oxidation in sediments, and here, reduced iron‐sulphide mineral phases could be the electron donors for this coupled process, as observed in laboratory studies (Bosch et al. 2012). Visual evidence of this process included Fe oxidation–reduction fronts observed during sediment collection, as well as micron‐sized framboid‐like minerals detected via SEM (Figure S1). Microbial sulphate reduction transcripts, which were prevalent in BC sediments, can initiate the conversion of SO_4_ and/or elemental S back into reduced iron‐sulphide phases, which is proposed to be a concomitant pathway in this system that returns oxidised S to a reduced form, which can then react with additional nitrate and perpetuate the cycle. Sediments at BC have lower oxidation–reduction potentials (ORP) with depth, well in the range expected for sulphate reduction (Falk et al. 2023). The detection of core‐active genera capable of S‐oxidation and N‐reduction genera in the summer and fall at BC, including *Thiobacillus*, *Sulfuritalea*, *Aquabacterium, Sideroxydans*, and *Dechloromonas*, supports these metabolic pathways (Guan et al. 2022; Wang et al. 2024; Watanabe et al. 2017; Xu et al. 2022). Studies within Big Creek have also observed decreasing N loads over time (Nelligan et al. 2021), which could be due in part to the differential expression of a nitrous oxide reductase (*nosZ*) involved in the conversion of N_2_O to N_2_, which completes the removal of NO‐species from the system and was hypothesised to be an active pathway in agricultural headwater sediments. Because N_2_O is a potent greenhouse gas, its removal through microbially mediated denitrification provides an added ecological benefit. Future research could focus on replicating the conditions found in Big Creek sediments to promote complete denitrification in constructed waterways, wetlands, or passive treatment systems. In summary, DNRA, as an ammonia‐producing/N‐reduction pathway, appears tightly coupled to S oxidation–reduction in the inorganic/chemical fertiliser‐impacted sediments, and *nosZ* may be responsible for NO_3_ removal trends in the system.

In terms of P, the high‐affinity periplasmic phosphate‐binding protein *PstS* showed higher expression at BC in the summer compared to expression at NC and SR. *Pst* genes can be expressed when P is limited (Harke et al. 2016; Hsieh and Wanner 2010); yet Big Creek exhibited high summer concentrations of SRP and TP relative to the other two sites. However, soluble P concentrations at Big Creek in the summer of 2020 (0.05 mg L^−1^) were lower than concentrations from early, middle, and late summer collections in 2019 (0.14, 0.09, and 0.11 mg L^−1^, respectively), thus the *PstS* response may be indicative of annual‐scale P availability trends at BC. In line with this, a low‐affinity inorganic phosphate transporter showed increased expression at BC in the fall when P concentrations were higher (0.08 mg L^−1^). Thus, microbial P metabolism genes observed at BC appear to reflect temporal P limitations and availability. The high abundance of the core‐active genus *OLB 12* at BC also supports these trends, as this taxon is known to be a phosphate‐accumulating organism (PAO) (Hei et al. 2022).

#### 4.3.2 Nissouri Creek—Microbial Functional Relationships to Manure Fertiliser Loads

At NC, higher NO_3_ concentrations in the water column and higher sediment TOC and labile P observed in a previous study are indicative of manure applications in the watershed (Falk et al. 2021; Jones et al. 2019). Despite NO_3_ being high, denitrification and DNRA genes were expressed less expressed than in BC and SR sediments, as were S‐oxidation genes. Rather, NC showed nitrosative stress and S‐assimilation genes with higher expression in the summer, when NO_3_ and SO_4_ concentrations were also higher. Nitrosative stress occurs in cells due to the accumulation of reactive nitrogen species (RNS) and is linked to excess N in the environment that is not removed by complete denitrification to N_2_ (Freedman et al. 2016; VanMensel et al. 2020). This aligns with the decreased expression of *nosZ* seen in NC sediments that microorganisms use to convert nitrous oxide to N_2_. In addition, hydroxylamine reductase (*hao*) was highly expressed in NC sediments during the summer, which oxidises ammonia into nitrogen intermediates (Soler‐Jofra et al. 2021), with the ammonia oxidising bacteria (AOB) *Nitrosomonas* identified as a core‐active taxon and highly abundant in RNA‐based amplicon analysis. *Nitrosomonas* may be partly responsible for the production of N intermediates that accumulate in the system that do not get removed via complete denitrification, triggering the high expression of nitrosative stress pathways in members of the microbial community. In addition, *Nitrosomonas* itself can be resistant to the effects of RNS, as reported in a previous study (Laloo et al. 2018), which would explain its high abundance in NC sediments. Nitrosative stress genes were expressed less expressed in the fall at NC, coinciding with lower N compound concentrations in the water column and the absence of *Nitrosomonas* as a core‐active taxon.

P metabolism trends at NC showed that *hppa* was highly expressed in both seasons, whilst P‐uptake and transport genes (including *SphS*, *PstA*, *PstB*, and *PstC*) increased in the fall, particularly in comparison to BC. This coincided with increases in SRP (750%) and TP (667%) water column concentrations in the fall at NC over summer, compared to increases of 60% and 19% at BC, respectively. *OLB12* and another PAO, *C. Accumulibacter*, were more abundant in NC sediment in the summer when P was more limited compared to the fall, which matched the trends at BC. The exact role of these PAOs in headwater sediments should be explored further, but it appears that they persist in agriculturally impacted sediments when P is seasonally low and possibly contribute to low available P by sequestering phosphate into cells, as observed in wastewater treatment biological P removal (BPR) studies (Rubio‐Rincón et al. 2017).

#### 4.3.3 Saugeen River—Microbial Functional Relationships at a Low‐Impact Reference Location

SR sediment microbial function and seasonal functional turnover were more similar to NC overall; however, there were also pathways that showed greater similarity to BC. Thus, SR proved to be an appropriate intermediate to compare divergent pathways of N, P, and S metabolism against the agriculturally impacted sites. *napA* and *nosZ* were consistent seasonally; however, ammonia processing and N‐fixation genes showed an increase relative to BC and NC in the fall. As external sources of TN are lower at the SR sampling site due to less intensive fertiliser applications, N‐fixation genes were hypothesised to be more expressed to supply bioavailable NH_4_^+^. For example, observations of small streams have shown a negative correlation between N fixation rates and nitrate‐N concentrations, with nitrate being more pronounced in agricultural streams (Caton et al. 2018). Our results support these conclusions, with N‐fixation genes also correlated with increased expression of ammonia assimilation/oxidation genes in the fall. Lower average available NO_3_ may also require microbial communities to utilise low‐affinity N‐reductases, such as *napA*, which showed high expression in SR sediments. In addition, SR also showed S‐oxidation expression trends similar to BC; thus, the coupled S‐oxidation/N‐reduction pathway hypothesised in BC sediments may also operate at SR. However, the primary taxon responsible for these transformations may be different. The core‐active genus *Sulfuritalea* showed the greater abundance in SR sediments, contrasting with *Aquabacterium* and *Thiobacillus* as the top contenders at BC, and is well recognised for its capacity for S‐oxidation using nitrate as the electron acceptor (Sperfeld et al. 2019).

In terms of P metabolism, *hppa* was highly expressed, like BC and NC. This demonstrates the universal importance of this gene, which encodes proteins key to cellular energy metabolism and ion transport, and future work may further explore *hppa* expression as a possible housekeeping gene for expression normalisation in metatranscriptomic studies.

In comparison to the agricultural sites, SR sediments exhibited elevated microbial diversity as indicated by the highest number of core‐active taxa detected (*n* = 68) and a high seasonal turnover in functionality. Although higher stream microbial diversity does not always correspond with decreased anthropogenic stress (Simonin et al. 2019), the detection of more active taxa via a DNA‐ and RNA‐based approach, along with the seasonal shift in function at SR, suggests a wider realised and fundamental niche, which can infer ecosystem resilience (Avila‐Jimenez et al. 2020; Girvan et al. 2005). Unique core‐active taxa at SR included *Ferritrophicum*, *Denitratisoma*, and *Sulfuricella*, and these taxa may be valuable microbial bioindicators of a healthy headwater sediment function.

## 5 Conclusions

Freshwater sediments in agriculturally impacted watersheds display distinct microbial functional and taxonomic patterns compared to less‐impacted reference sites, highlighting the influence of chronic nutrient loads on microbial biogeochemical cycling. Using RNA‐ and DNA‐based sequencing alongside metatranscriptomics, this study provides evidence that RNA‐derived taxa align more strongly with N, P, and S metabolic functions than DNA‐derived taxa, emphasising the utility of RNA for capturing active microbial processes. Site‐specific trends revealed significant differences in nutrient metabolism across impacted sites, which aligned with watershed fertiliser characteristics as well as temporal changes. Big Creek, characterised by inorganic fertiliser amendment, exhibited tightly coupled nitrate reduction and sulphur oxidation pathways, whilst Nissouri Creek, influenced more by manure applications, showed enhanced nitrosative stress and sulphur assimilation processes, with low expression of *nosZ* genes. In contrast, the reference site, Saugeen River, demonstrated intermediate functional turnover, with evidence of greater nitrogen fixation to supply bioavailable N to the system, and higher diversity in RNA/DNA‐leveraged core‐active microbial taxa, indicative of a healthier ecosystem. Seasonal variation, particularly in the fall, amplified differences in microbial activity and core‐taxa turnover, underscoring the importance of incorporating temporal dynamics in assessments of nutrient‐driven microbial transformations.

With respect to our hypotheses:

1. RNA‐based taxa data correlated more strongly with metatranscriptomic datasets than DNA‐based taxa.
2. Agriculturally impacted sites differed from the reference site in terms of sediment microbial function, but inorganic and manure‐affected watershed sites showed greater differences to each other.
3. Phosphorus uptake and metabolism gene expression patterns in sediments were complex and potentially responded to temporal P limitations rather than the general expectation of P loads to watersheds. *nosZ* was highly expressed at the inorganic‐fertiliser‐impacted and reference sites, but not at the manure‐impacted site, suggesting a short circuit in N removal at the latter.
4. N‐fixation appeared more pronounced at the low‐impact reference site as a means of supplying bioavailable nitrogen.

These findings suggest that non‐point nutrient anthropogenic stressors not only alter microbial function but also drive site‐specific adaptations, providing critical insights for monitoring and managing nutrient impacts in freshwater ecosystems.

## Author Contributions

**Nicholas W. Falk:** conceptualization, investigation, writing – original draft, methodology, visualization, writing – review and editing, formal analysis, data curation. **Ian G. Droppo:** conceptualization, investigation, writing – review and editing, methodology, formal analysis, project administration, resources, supervision. **Ken G. Drouillard:** conceptualization, investigation, funding acquisition, methodology, writing – review and editing, formal analysis, project administration, resources, supervision. **Christopher G. Weisener:** supervision, resources, project administration, formal analysis, writing – review and editing, methodology, conceptualization, investigation, funding acquisition.

## Ethics Statement

The authors have nothing to report.

## Consent

All authors agreed with the content and that all gave explicit consent to submit and that they obtained consent from the responsible authorities at the institute/organisation where the work has been carried out.

## Conflicts of Interest

The authors declare no conflicts of interest.

## Supporting information


**Figure S1:** Evidence of Fe oxidation–reduction fronts in Big Creek bed sediments. (A) Surface sediment exhibiting Fe‐oxidation characteristics. (B) Framboidal‐like mineral phase in surface sediments consistent with reduced FeS habit. Sediment was placed on an SEM sample stub and air dried, and then examined using a FEI Quanta 200F ESEM under low vacuum.

**Table S1:** Big Creek and Nissouti Creek agricultural characteristics.
**Table S2:** 16S Amplicon sample metadata.
**Table S3:** Bed sediment Metatranscriptomic sample details.
**Tabls S4**. DESeq2 pairwise site comparisons for summer functions.
**Tabls S5**. DESeq2 pairwise site comparisons for fall functions.
**Tabls S6**. DESeq2 pairwise site comparisons for season all sites.
**Tabls S7**. Lefse three‐way site comparisons for summer for significant functions identified via DESeq2.
**Tabls S8**. Lefse three‐way site comparisons for fall for significant functions identified via DESeq2.

## Data Availability

The data that supports the findings of this study are available in the Supporting Information of this article.
