# Supplementary material for: Combined DNA/RNA Amplicon Sequencing and Metatranscriptomics Reveals Microbial‐Driven Nutrient Transformations and Core Taxa in Agriculturally Impacted Sediments
Source: Environ Microbiol Rep. 2025 Sep 27;17(5):e70205. doi: 10.1111/1758-2229.70205 (PMC12475508; doi:10.1111/1758-2229.70205)
Supplement: Supplementary file 1 — Figure S1: Evidence of Fe oxidation–reduction fronts in Big Creek bed sediments. (A) Surface sediment exhibiting Fe‐oxidation characteristics. (B) Framboidal‐like mineral phase in surface sediments consistent with reduced FeS habit. Sediment was placed on an SEM sample stub and air dried, and then examined using a FEI Quanta 200F ESEM under low vacuum. [file EMI4-17-e70205-s002.docx]

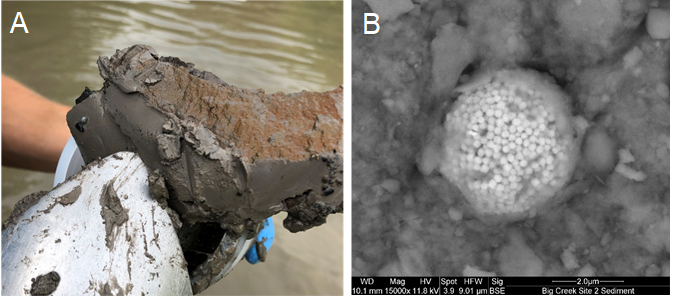


Figure S1: Evidence of Fe oxidation-reduction fronts in Big Creek bed sediments. A) Surface sediment exhibiting Fe-oxidation characteristics. B) Framboidal-like mineral phase in surface sediments consistent with reduced FeS habit. Sediment was placed on an SEM sample stub and air dried, and then examined using a FEI Quanta 200F ESEM under low vacuum.
